# The BioRef Infrastructure, a Framework for Real-Time, Federated, Privacy-Preserving, and Personalized Reference Intervals: Design, Development, and Application

**DOI:** 10.2196/47254

**Published:** 2023-10-18

**Authors:** Tobias Ueli Blatter, Harald Witte, Jules Fasquelle-Lopez, Christos Theodoros Nakas, Jean Louis Raisaro, Alexander Benedikt Leichtle

**Affiliations:** 1 University Institute of Clinical Chemistry University Hospital Bern Bern Switzerland; 2 Graduate School for Health Sciences University of Bern Bern Switzerland; 3 Biomedical Data Science Center University Hospital Lausanne Lausanne Switzerland; 4 Laboratory of Biometry University of Thessaly Volos Greece; 5 Center for Artificial Intelligence in Medicine University of Bern Bern Switzerland

**Keywords:** personalized health, laboratory medicine, reference interval, research infrastructure, sensitive data, confidential data, data security, differential privacy, precision medicine

## Abstract

**Background:**

Reference intervals (RIs) for patient test results are in standard use across many medical disciplines, allowing physicians to identify measurements indicating potentially pathological states with relative ease. The process of inferring cohort-specific RIs is, however, often ignored because of the high costs and cumbersome efforts associated with it. Sophisticated analysis tools are required to automatically infer relevant and locally specific RIs directly from routine laboratory data. These tools would effectively connect clinical laboratory databases to physicians and provide personalized target ranges for the respective cohort population.

**Objective:**

This study aims to describe the BioRef infrastructure, a multicentric governance and IT framework for the estimation and assessment of patient group–specific RIs from routine clinical laboratory data using an innovative decentralized data-sharing approach and a sophisticated, clinically oriented graphical user interface for data analysis.

**Methods:**

A common governance agreement and interoperability standards have been established, allowing the harmonization of multidimensional laboratory measurements from multiple clinical databases into a unified “big data” resource. International coding systems, such as the International Classification of Diseases, Tenth Revision (ICD-10); unique identifiers for medical devices from the Global Unique Device Identification Database; type identifiers from the Global Medical Device Nomenclature; and a universal transfer logic, such as the Resource Description Framework (RDF), are used to align the routine laboratory data of each data provider for use within the BioRef framework. With a decentralized data-sharing approach, the BioRef data can be evaluated by end users from each cohort site following a strict “no copy, no move” principle, that is, only data aggregates for the intercohort analysis of target ranges are exchanged.

**Results:**

The TI4Health distributed and secure analytics system was used to implement the proposed federated and privacy-preserving approach and comply with the limitations applied to sensitive patient data. Under the BioRef interoperability consensus, clinical partners enable the computation of RIs via the TI4Health graphical user interface for query without exposing the underlying raw data. The interface was developed for use by physicians and clinical laboratory specialists and allows intuitive and interactive data stratification by patient factors (age, sex, and personal medical history) as well as laboratory analysis determinants (device, analyzer, and test kit identifier). This consolidated effort enables the creation of extremely detailed and patient group–specific queries, allowing the generation of individualized, covariate-adjusted RIs on the fly.

**Conclusions:**

With the BioRef-TI4Health infrastructure, a framework for clinical physicians and researchers to define precise RIs immediately in a convenient, privacy-preserving, and reproducible manner has been implemented, promoting a vital part of practicing precision medicine while streamlining compliance and avoiding transfers of raw patient data. This new approach can provide a crucial update on RIs and improve patient care for personalized medicine.

## Introduction

### Reference Intervals in Clinical Diagnostics

The use of blood tests is a cornerstone of disease diagnosis and health assessment in clinical medicine. When clinicians try to assess the health status of patients, they heavily rely on laboratory tests and population-based measures such as the reference interval (RI). In their core concept, RIs enclose a fixed range of values from a predefined reference population (eg, 95%), and it has long been established that they are effective in clinical use as long as they are precise and accurate [1-4]. Clinical laboratories must independently establish and periodically verify their RIs in use through admissible guidelines [5]. The widely used guideline EP28-A3c developed by the Clinical and Laboratory Standards Institute (CLSI) and the International Federation of Clinical Chemistry (IFCC) states that RIs should be estimated from cohort-relevant reference populations, where not only patient group–specific covariates such as age, biological sex, ethnicity, and region are considered but also differences in preanalytical factors are accounted for [6]. The process is cumbersome, costly, and often beyond the scope and possibilities of many independently operating laboratories: cohort-specific analyses require stratification by a specific combination of the above-mentioned covariates. Therefore, these analyses are frequently limited to small sample sizes owing to a lack of available data [7,8].

In addition, most studies have been conducted with very lenient inclusion and exclusion criteria owing to a missing overarching definition of “health,” covering both the normative aspects (well-being and functioning) and more descriptive aspects of health evaluation (test result assessment). This hinders the comparability of the generated RIs [9]. A common classification framework to define the health status of the included participants based on predetermined medical conditions is required. In this context, the International Classification of Diseases (ICD) is a commonly used coding system to help represent nuanced diseases to broader morbidities [10]. Inference of RIs with the exclusion or inclusion of specific combinations of diseases (representing the health status of the patient) might enable the personalization of the diagnostic use and provide target ranges that allow the interpretation of the results based on the specific condition of the individual patient [11]. This would essentially allow the creation of RIs as “expectation ranges” for “digital twins,” that is, patients who share similarities with the patient under observation but do not have a specific disease. Particularly for older patients or patients with multiple morbidities, this comparison is seemingly more appropriate, as the concept of a “healthy reference” is inherently unattainable for these populations [12]. In addition, international efforts, such as that of the IFCC’s Task Force on Global Reference Interval Database, aim at generating resources for RIs at a global scale [13].

### Harmonized RIs

The aforementioned limitations can be overcome through multicenter collaborative RI studies, where standardized protocols help derive *harmonized RIs* at a national level by pooling the appropriate number of patients from multiple cohorts [14]. Such standardization requires clear classification systems, for example, for the nomenclature, terminology, units, and formats used, to ensure the reproducibility of all the steps of the complete laboratory testing procedure, possibly for international application [15,16]. This is an ongoing global process, as laboratories in Europe [17-22], Africa [23-25], North America [26], Asia [27-29] and Australia [30] aim at deriving nation-specific RIs through multicenter studies.

The broader introduction of locally inferred RIs from harmonized data sets has not been observed across the board in clinical laboratories [31,32]. Endeavors estimating patient group–specific RIs from electronic health records have shown successful results yet remain sparse [33]. This is mostly due to a lack of sophisticated analysis tools connecting laboratory databases, where multidimensional data are readily available, to physicians in need of clinically relevant RIs. For each standardization effort, clinical physicians or laboratory specialists have to go through significant administrative burden, as they realign the laboratory data for each RI study individually.

### The Need for a Streamlined Research Data-Sharing Infrastructure

Switzerland has one of the most restrictive laws surrounding the nature of the collection and sharing of identifying information and personal data, including health data (all referred to as *sensitive data*). Sensitive data require careful governance, covered by the Swiss Federal Act on Data Protection 1992 (article 3c) [34]. The processing of *sensitive data* for research is further referenced in the Human Research Act (Federal law 810.30). Unless clinical research data are anonymized, studies require the approval of an ethics committee. Owing to these prerequisites, intercohort data sharing mandates a Data Transfer and Use Agreement between the data provider and the recipient before any sensitive data can be exchanged. Such a practice is common in many other countries as well and causes significant administrative overhead, at times rendering potential stakeholders hesitant to join multiparty research projects. In Switzerland, a national IT environment for sensitive research data, the BioMedIT infrastructure, was established to ensure a backbone for the secure transfer, storage, management, and processing of confidential data [35]. Despite all the progress achieved by this streamlined infrastructure, the hurdle for nationwide data pooling is still relatively high. A recent effort to establish a Swiss multicohort resource in pharmacogenetics has been documented to take up to a year for just setting up the legal and scientific framework [36]. Novel privacy-preserving data exchange and data processing options or platforms could alleviate the regulatory burden imposed on multicohort projects.

### The BioRef Vision

The need for an intercohort data-sharing infrastructure that allows a more streamlined process for individual researchers accessing the relevant reference populations and estimating applicable RIs is apparent. The BioRef rationale is to establish an infrastructure that allows the creation of precise RIs from pooled data based on an interoperable semantic framework. Instead of placing the responsibility for data interoperability and aggregation on individual laboratory specialists, establishing an opportunity for clinical laboratories to conveniently and reproducibly check whether their standard RIs apply to their patient populations is important. It should be an essential part of precision medicine practiced today. Ideally, this involves web applications with easily accessible graphical user interfaces (GUIs) that allow the recurrent aggregation of patient data in an accreditation-proof manner and the transfer of the aggregated data from all partners to the interested laboratory specialists (end users). The BioRef initiative relies on a federated and privacy-preserving approach for secure analytics based on multiparty homomorphic encryption [37]. Combining the data of multiple providers broadens a project’s data basis, that is, it results in higher data coverage. Moreover, it increases the chances of gaining insights from rare patient profiles. Data from diverse sources, however, tend to be heterogeneous, which makes it more difficult to leverage and extract interoperable insights. Our federated approach is implemented in the software system *TI4Health,* the commercial version of its open-source predecessor *Medco*, a secure system for privacy-preserving federated data exploration and analyses based on advanced privacy-enhancing technologies [38,39]. With this, data remain on the premises and under the full control of the participating institutions. Only the aggregated result of the requested computation is released over the entire distributed virtual database to an authorized user [40]. As RIs are essentially a population aggregate, systems using aggregate data, such as *TI4Health*, reduce the risk of reidentification of patients owing to the potentially imperfect deidentification of clinical data.

## Methods

### BioRef Governance and Semantic Interoperability

The parties involved in the *Swiss BioRef* project have formed a multicenter research consortium, the *BioRef consortium*, which currently consists of 4 major cohort sources in Switzerland: the University Hospital Bern (“Inselspital”), the University Children’s Hospital Zürich (Kinderspital Zürich), Swiss Paraplegic Research, and the University Hospital Lausanne (Centre Hospitalier Universitaire Vaudois [“CHUV”]). The consortium agreement covers multiple aspects of this collaborative effort, including data governance, data delivery, and the required network infrastructure. Participating institutions agreed to contribute their data by making them accessible via a decentralized platform and transferring them to a centralized trusted data host for a validation approach.

The key component for creating a sustainable and an expandable infrastructure is the definition of intercohort concepts regarding semantic interoperability, availability, dimensionality, and quality of the data provided by different cohorts. It is vital that each clinical partner involved is willing to process the data to adhere to harmonized and interoperable standards for data encoding, including Logical Observation Identifiers Names and Codes (LOINC [41]; for analyses); the ICD, Tenth Revision (ICD-10; for diagnoses); and the Anatomical Therapeutic Chemical classification system (for medication). As a preferred semantics and data representation logic of the Swiss Personalized Health Network (SPHN), the Resource Description Framework was chosen, with the underlying *BioRef* ontology based on the SPHN ontology (release 2021-2) [42].

### BioRef Data Recruitment

Data from each contributing cohort consist of quantitative laboratory test results (“measurements”) from 46 frequent laboratory variables uniquely defined by LOINC encoding (Multimedia Appendix 1). Data extraction from the clinical data warehouses and data deidentification (removal of direct identifiers) were exclusively carried out locally by the data scientists of each consortium partner. Data were included only if the patients provided written consent. Routine clinical laboratory data of inpatients were included if at least one LOINC-coded laboratory analysis of interest was performed during the administrative case (admission) and at least one diagnosis (ICD-10-German Modification [GM] coded) was recorded after the administrative case was closed. Notably, inpatients of Swiss hospitals always have at least one ICD-10 diagnosis assigned for billing purposes. To limit the bias caused by repeated measurements, only the first measurement of each LOINC of interest per administrative case was included in each contributing cohort data set. This first value out of a series of values is the least influenced by potential therapeutic measures. Such a practice is in line with previous cohort-specific RI studies [32,43,44]. Each laboratory measurement is currently enriched with patient record information from the clinical data warehouses of the involved hospitals, including age, sex, and the 5 most relevant previously established diagnoses using the ICD-10-GM codes [10]. Age is provided in years with a precision of 3 decimal places for patients aged <18 years and as whole numbers (integer) for patients aged ≥18 years. Attributes for sex are assigned from the set “female, male, other, or unknown,” as predefined in many hospital information systems. The diagnoses used in the BioRef data set represent those recorded at the discharge of the patient. The “relevance” of diagnoses follows the guidelines of the Swiss Federal Office of Public Health, that is, diagnoses represent the so-called «billing diagnoses» used by hospitals for reimbursement from health insurances. In general, the effort required and severity of a diagnosis are considered to correlate. This approach is widely and uniformly used across hospitals. Furthermore, information on the generation of the measurement (analytical factors) is included as linked metadata. This specifies the analyzer and the test kit and reagent used through the unique identifiers for medical devices from the Global Unique Device Identification Database [45] as well as the type identifiers from the Global Medical Device Nomenclature [46]. These additional metadata help overcome the sparsity of information associated with LOINCs with respect to the applied method. Data made available to the project under the consortium agreement span the time frame from June 2014 to February 2023.

### Ethical Considerations

This study received an ethics waiver from the cantonal ethics committee of Bern (Business Administration System for Ethics Committees; BASEC-Nr: Req-2020-00630). The platform was initialized using a bulk data load. It is updated on a regular basis, although there is no particular pressure for frequent updates.

### BioRef-Federated Analytics Approach

On the basis of common data semantics and under a common contractual architecture, the *Swiss BioRef* project relies on a *decentralized approach* for multicohort data pooling to align the BioRef data independently of the available IT resources at each cohort site. Consortium partners compile their data set on their own accord while maintaining full control over the data-sharing process.

The decentralized mechanism underpinning the BioRef infrastructure is based on a privacy-preserving protocol that uses a multiparty homomorphic encryption scheme and obfuscation techniques to allow privacy-preserving federated querying with secure aggregation [37]. It relies on a fully decentralized peer-to-peer infrastructure with no central node, enabling the processing of sensitive data under homomorphic encryption and release of results aggregated across all participating sites [37]. This *federated approach* follows a strict “no copy, no move” principle, where clinical data do not leave the local site’s database, and only encrypted aggregates are exchanged and further processed between different nodes, always under encryption. This information exchange system requires a minimum of IT components deployed locally. If a data holder is unable to provide the required infrastructure and personnel, node instances can also be installed off-premises within a trusted IT infrastructure.

A proven *centralized approach* involving a trusted data host system jointly used by the data providers was also implemented as a baseline reference for the verification and validation of the federated approach. This mechanism relies on the existing secure BioMedIT network set up by the Swiss Institute of Bioinformatics: data from *BioRef* consortium partners are locally collected, encrypted on site with traditional public key cryptography by the data providers, and subsequently securely transferred to a highly restricted project space within the *BioMedIT* network [47].

### Statistical Analysis

#### Data Preprocessing

The *BioRef* platform allows the user to interactively design a cohort for querying an underlying “big data” source. To tidy up the input data, a preliminary data cleaning step was introduced to remove measurements from the raw data set that had missing or clearly erroneous entries, including occasional negative values where an analysis does not allow them or ICD-10-GM codes not in use as of May 2022. Furthermore, *outlier detection* was introduced as the first step of the interactive RI inference algorithms to limit the influence of extreme values *(outliers)* on the statistical inference. An outlier is informally defined as a data point that significantly deviates from most of the available data [48]. A 3-sigma range (based on the query sample’s mean and SD) was identified to generally detect data points from the harmonized multicohort data set that most likely stemmed from the patient population under consideration. Values outside this 3-sigma range were flagged and removed.

#### RI Calculation Methods

The gold standard for inferring the RI has long been *direct methodology*, where test results are sampled from a homogeneous and presumably healthy reference population, and the 2.5th and 97.5th percentiles of the obtained sample are determined [49]. Owing to cohort-specific definitions of health, it is often difficult to harmonize RIs across different patient groups. *Indirect methods* of RI estimation offer a way to address this limitation [50]. Indirect methods sample and weight test results from a mixed clinical population, including both physiological and pathological test results from routine patient care (general admission to the hospital) [51]. In the context of *BioRef*, both *direct* and *indirect* RI inference methods (with parametric and nonparametric estimations) were adjusted to be fully automated. Following the official recommendation, the standard nonparametric quantile estimation method was implemented [6]. Various factors influence the precision and consistency of the inferred RIs, such as measurement variability; sample size; and, in general, the underlying reference value distribution. For skewed reference distributions exhibiting a single peak, an adaptation of the robust quantile estimator method was implemented [52]. This method contains a parametric Box-Cox transformation step and uses a biweight quantile estimator to calculate the appropriate ranks [53,54]. For analyte distributions that exhibit multiple peaks, an iterative method was proposed to resolve the Gaussian main mode from the distribution mixture [55]. This involves iteratively trimming the overall distribution, assuming a Gaussian distribution in the central region, and subsequently readjusting the SD to account for the trimmed data until convergence. Alternatively, a modified and fully automated Bhattacharya procedure was implemented, where binned data are used to decompose a distribution into Gaussian subcomponents [56]. The developed methods underwent internal testing to ensure their robustness toward outliers and ability to handle varying degrees of skewness. Using bootstrapping techniques, it is possible to estimate the precision of all the implemented methods by generating 90% CIs for the RI boundaries. These CIs simultaneously reflect the precision of the pulled analyte data aggregate and the suitability of the RI methodology in the light of the overall estimation.

#### Power

The *BioRef* analytics platform does not estimate new RIs for reference samples of <120 patients, thereby considering the general statistical limitations of RI estimation in accordance with the CLSI guidelines [6]. This means that cohorts of interest with >120 individuals are sufficiently represented. An option for validating the existing RIs with population sizes <120 patients in line with the CLSI validation guidelines is planned for a future release.

### Privacy Protection

With the underlying “big data” source, it is necessary to implement mechanisms that ensure end-to-end privacy protection when allowing end users to highly stratify the patient population. The values from a patient query for each cohort are securely aggregated under multiparty homomorphic encryption across all cohorts in a joint frequency table (for histogram building), which can be decrypted only by authorized users. Thus, both patient-level information and local aggregates are protected. Whereas the former never leaves the data holder infrastructure, the latter is always processed under encryption. With the limitation of a minimum of 120 patients and a rounded bin size width, the potential for individual reidentification of patients from the decrypted frequency table can be hindered.

When patient-level data are centralized into the *BioRef* secure project space on the *BioMedIT* infrastructure for validation purposes, further deidentification measures are implemented to minimize reidentification risks due to potential data leakages. Particularly, linkages between patients, administrative cases, and measurements had to be removed by the contributing cohorts after local data extraction (“local deidentification”). Measurements in the centralized *BioRef* data set for validation are, therefore, not linked at any level.

## Results

### BioRef Architecture and Data Contributions

**Figure 1:**
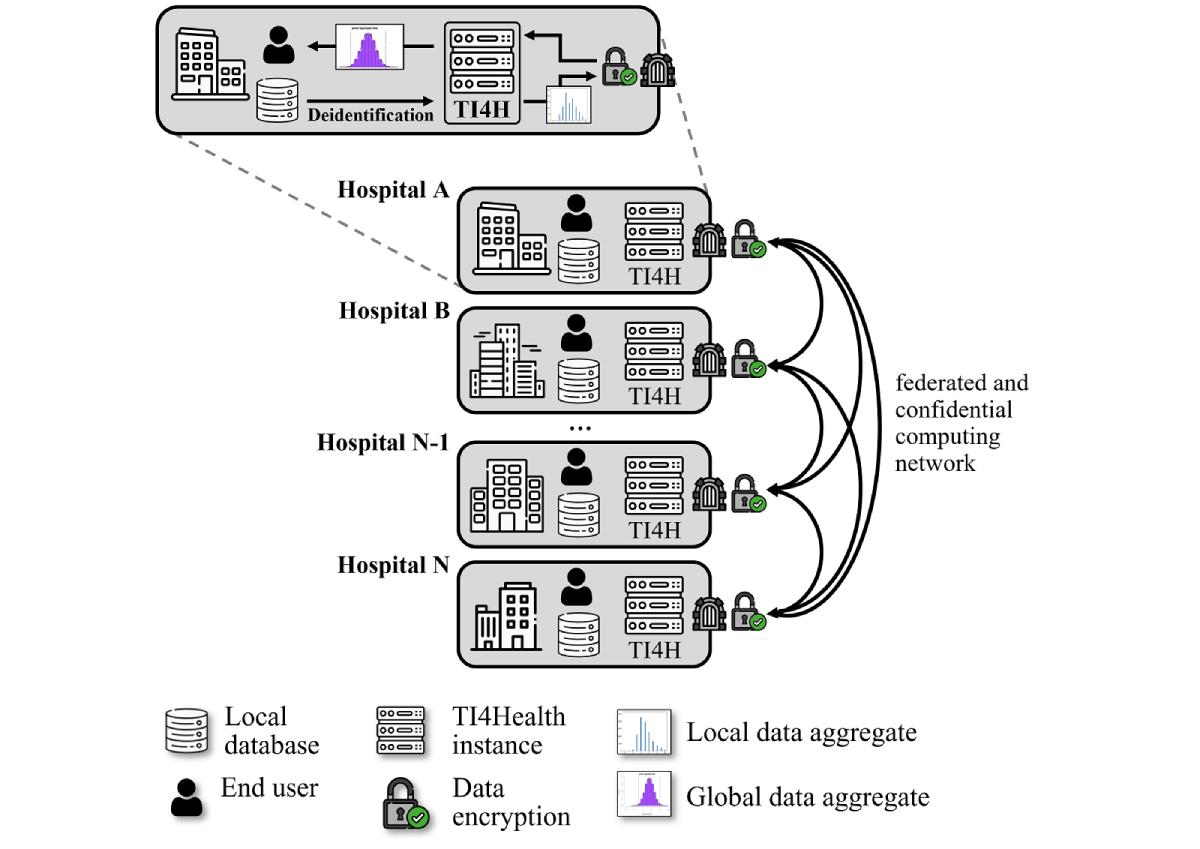
Illustration of the BioRef federated analytics infrastructure. In the decentralized approach, data is de-identified on site by the individual data providers of the consortium (hospital A, hospital B, ...) and uploaded to the on-premise TI4Health instance. Data are analyzed via the federated confidential computing network without any raw data of the consortium members being revealed.

Currently, the *BioRef* analytics platform is deployed with harmonized and interoperable data contributions from all BioRef consortium members. The use of the *TI4Health* architecture allows patient-level data to stay on site at each participating institution regardless of its location, and aggregated frequency tables are computed under multiparty homomorphic encryption, thus ensuring end-to-end data protection (Figure 1). This enables the aggregation of clinical data in a unified manner to create a comprehensive database. User-requested patient queries initiated via the GUI are relayed to the *TI4Health* instances, which constitute a distributed network for federated confidential computing. Homomorphically encrypted local data aggregates are then exchanged among the network partners to form the global data aggregate. RI computation is carried out by the front end of *Swiss BioRef TI4Health*, which returns the global aggregate result to the user (Figure 1). Notably, the raw data of the data providers are never shared (the “no copy, no move” principle).

Data from the contributing cohorts consisted of quantitative results from >40 frequently requested key laboratory tests, including analyses from clinical chemistry, hematology, point-of-care testing, and coagulation. These pooled standardized data (approximately 9 million measurements) constituted the multicohort database available on the *BioRef* platform (Table 1). It currently entails not only data from 2 university hospitals (Inselspital and CHUV) reflecting a broad variety of patients from the general population but also more specific data of patient groups in need of particular care, specifically children (University Children’s Hospital Zürich) and patients with physical disabilities (Swiss Paraplegic Research). Together, this multifaceted, highly standardized data set represents a rich “big data” source ready for further analyses, including end user–driven patient query stratification for the definition of specific RIs.

**Table 1:** Data contributions of the individual data providers for the BioRef infrastructure as of the time of publication.

		Inselspital	KiSpi^a^	Swiss Paraplegic Research	CHUV^b^	Total
Measurements, n		6,793,937	454,155	35,271	1,708,454	8,991,817
Unique patients, n		205,437	17,179	887	56,809	N/A^c^
**Patient sex, n (%)^d^**						
	Female	100,612 (49)	7695 (44.8)	278 (31.3)	30,739 (54.1)	N/A
	Male	104,825 (51)	9484 (55.2)	609 (68.7)	26,070 (45.9)	N/A
Patient age (years), median (IQR)		57 (32-73)	4.51 (0.76-10.97)	58 (42-71)	58 (39-73)	N/A
Administrative cases^e^		363,912	28,393	887	132,344	525,531
Unique LOINC^f^		39	37	33	23	46
Time span^g^		June 2014 to February 2023	April 2014 to May 2022	Up to March 2022	January 2020 to December 2022	N/A

^a^KiSpi: “Kinderspital Zürich,” University Children’s Hospital Zurich.

^b^CHUV: “Centre Hospitalier Universitaire Vaudois,” University Hospital Lausanne.

^c^N/A: not applicable.

^d^No nonbinary patients were reported at the time of publication.

^e^Admissions.

^f^LOINC: Logical Observation Identifiers Names and Codes.

^g^Time span during which the measurements were collected.

### BioRef-Federated Analytics Platform

**Figure 2:**
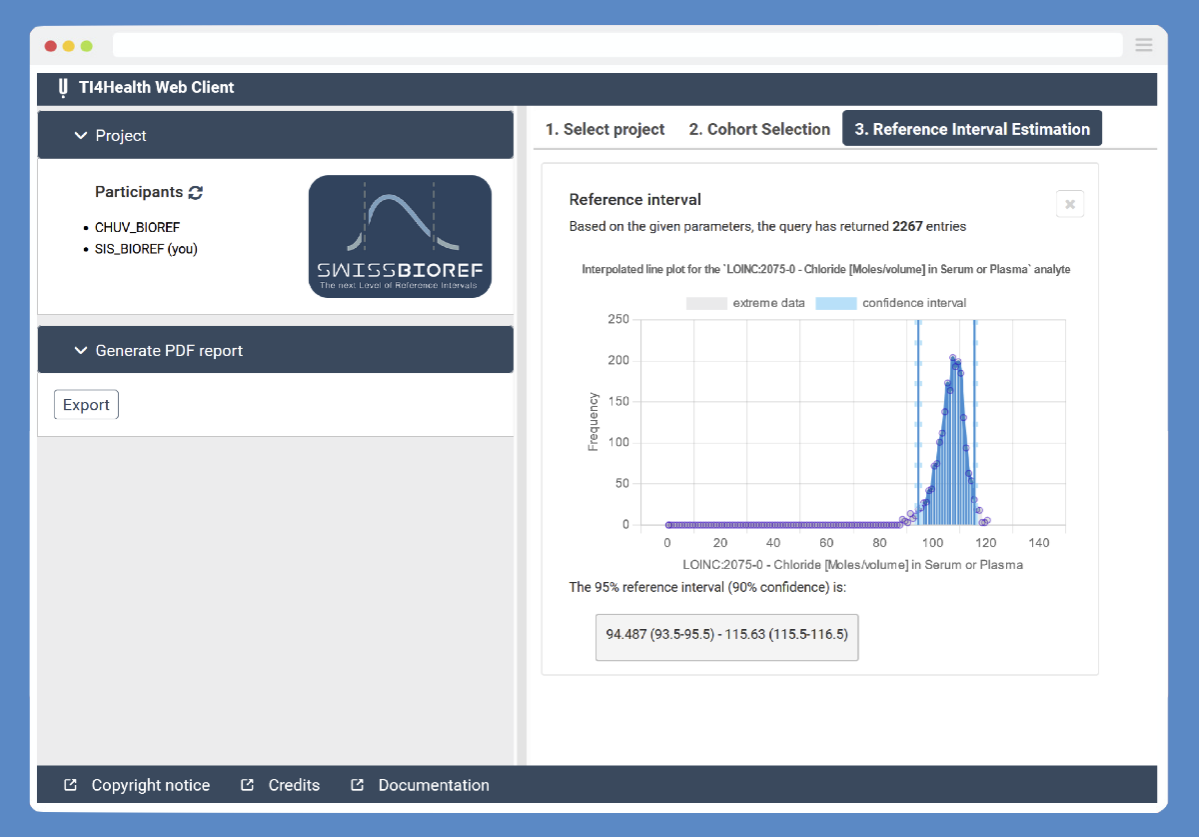
Graphical user interface of the Swiss BioRef TI4Health webclient. The web application shows the estimates for reference intervals and an accompanying histogram for “chloride in serum or plasma” (LOINC 2075-0) for a female patient cohort aged 55 to 60 years as an exemplary query.

The decentralized privacy-preserving approach was built on the *TI4Health* operational system (“Swiss BioRef TI4Health”; Figure 2). The extended *TI4Health* system in the context of the *BioRef* platform contains (1) the Informatics for Integrating Biology and the Bedside (i2b2) common data model, which is one of the most widely used data models for storing observational longitudinal clinical data and related metadata and is currently implemented in >300 hospitals worldwide and used by most of the Swiss university hospitals, running in a Postgres database [53]; (2) the *TI4Health* distributed backend; (3) a RESTful application programming interface; and (4) a customized *TI4Health* web client front end (Figure 2).

On the backend, *TI4Health* is built via a separate but modular approach, in which the front end query system never directly accesses the unencrypted data stored in the i2b2 data model but communicates only with the backend through the RESTful application programming interface. Once a request is received, the *TI4Health* backend module forwards it to an i2b2 connector for local data preprocessing and then starts the secure multiparty homomorphic encryption–based distributed aggregation protocol that involves all the other nodes in the network. The encryption protocols used in *TI4Health* are based on the Lattigo homomorphic encryption library [57]. The data were translated from the Resource Description Framework to the i2b2 format using a data converter module, which was developed during the course of the project [53].

On the front end, the *TI4Health* web client is the user-facing web application based on *Glowing Bear*, an open-source web-based GUI for cohort selection and analysis [58]. For BioRef, the *Glowing Bear* interface was tailored to allow the generation and visualization of precise RIs using an IFCC- and a CLSI-suggested method for nonparametric RI estimation. More specifically, the *BioRef* GUI allows for interactively setting and executing patient queries based on the covariates and running the statistical inference method on the returned measurements from the client side (Figure 2). It allows setting the patient’s “age” and “sex” as possible stratification variables and including not only diseases or risk factors, such as high blood pressure and diabetes (using the respective ICD-10 code) but also analysis-specific metainformation such as analyzer, test kit, and vendor information.

### Centralized Validation Platform

**Figure 3:**
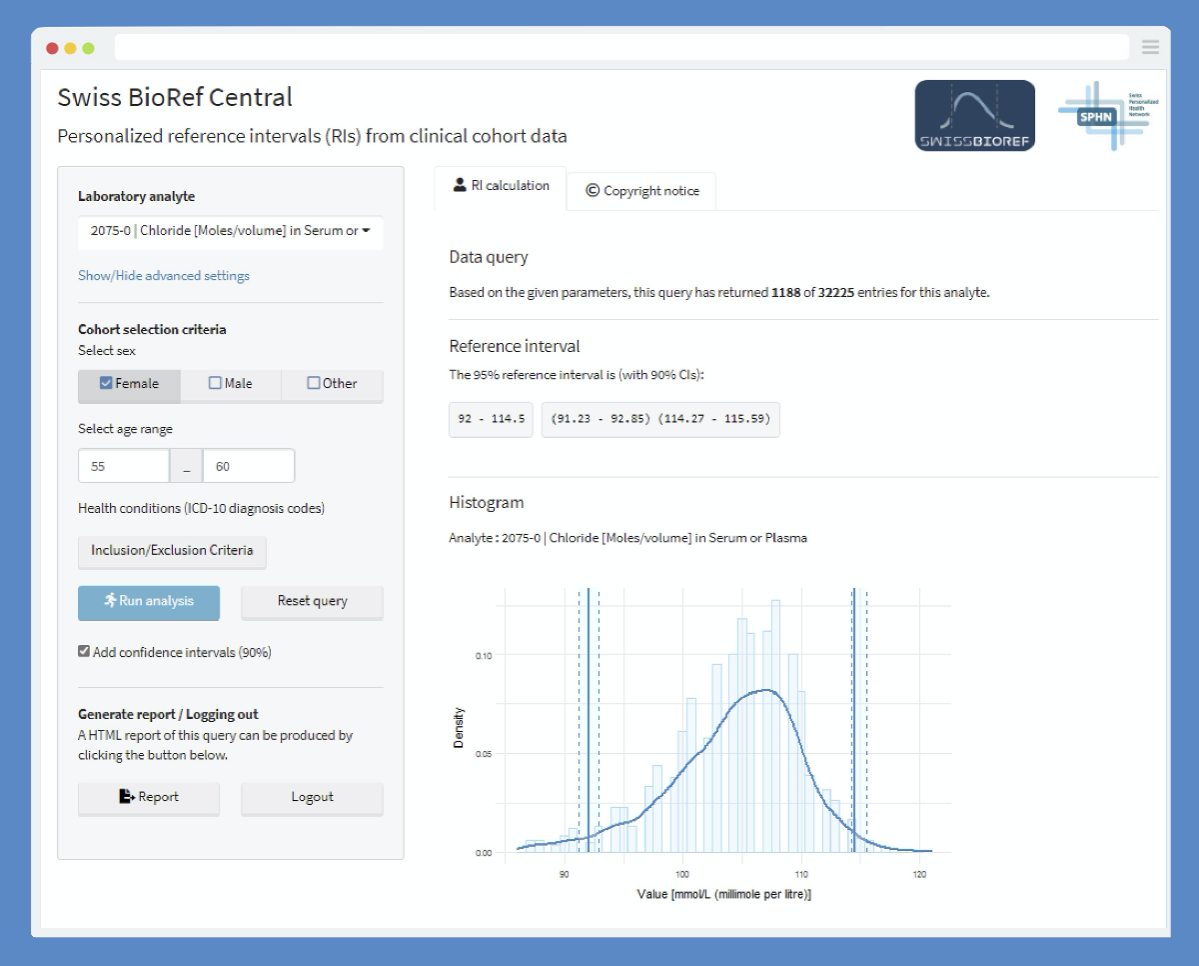
Screenshot of the Swiss BioRef Central interface. Graphical user interface of the Swiss BioRef Central web application. The web applications show the estimates for reference intervals for “chloride in serum or plasma” (LOINC 2075-0) for a female patient cohort aged 55 to 60 years as an exemplary query.

A separate central validation platform (*Swiss BioRef Central*) was set up on the secure *BioMedIT* infrastructure for method development, benchmarking, and ensuring the correctness of multicohort federated and encrypted analyses (Figure 3). Such a platform enables performance and usability comparisons between decentralized and centralized approaches and the testing of the accuracy of the statistical methods in inferring precise RIs from multicohort resources. The platform offers both direct (IFCC and CLSI approved) and indirect (using newer data mining techniques) methods for the inference of RIs.

**Figure 4:**
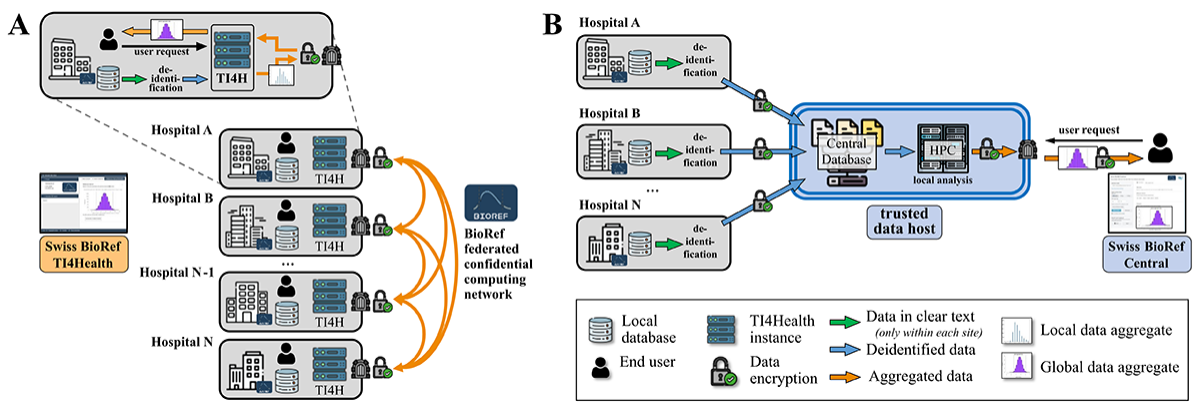
BioRef platform architecture. Side-by-side comparison of (A) the BioRef decentralized privacy-preserving platform using federate confidential computation and decentralized data linking and (B) the centralized validation platform that enables evaluation from a centralized data pool located within a trusted data host system. Both infrastructures offer their own web applications capable of inferring highly relevant reference intervals from their respective linked data sources.

This reference platform was built on R Shiny (R Studio, Inc), an operative extension of the R programming language into web application development to allow reactive and interactive data analyses [56]. It runs fully dockerized on a virtual machine with full access to the centralized deidentified data stored in CSV format (Figure 4B). The web traffic of *Swiss BioRef Central* was implemented behind a reverse proxy layer in the application architecture. This hides server traffic and communication to the front end of the application, which further reduces the risk of exposing sensitive information to the front end.

### Targeted RIs for Diagnostic Application

**Figure 5:**
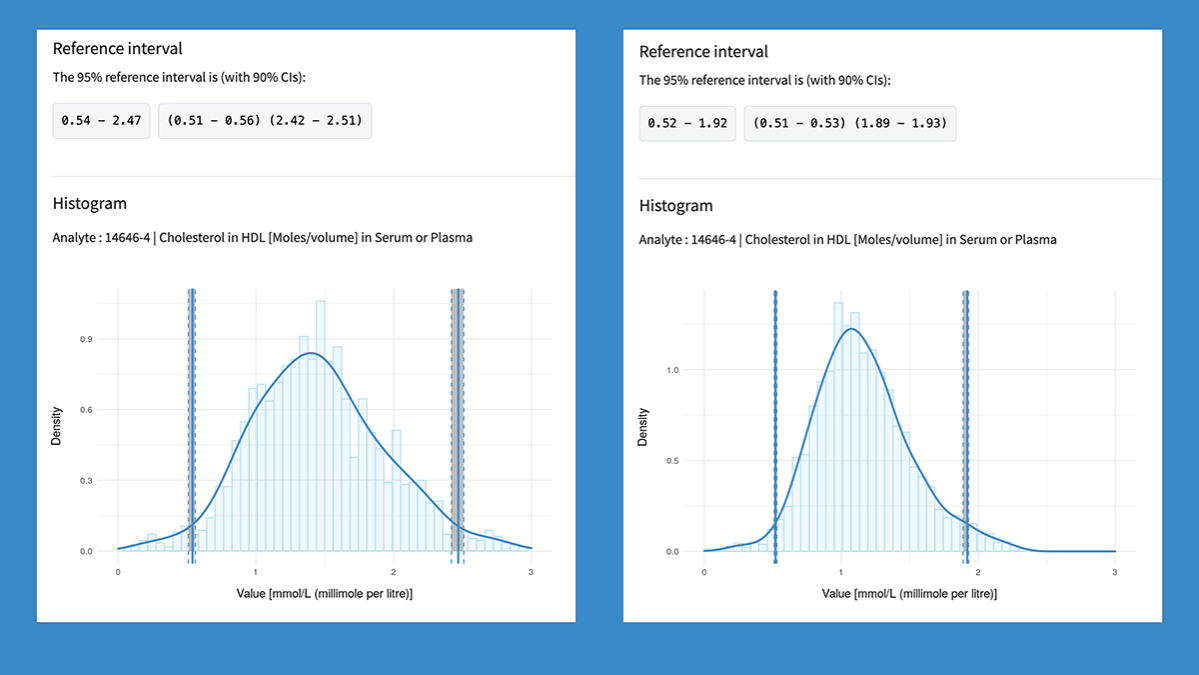
Personalized ranges for high-density lipoprotein cholesterol (in mmol/L). Estimated reference intervals for “cholesterol in HDL [moles/volume] in serum or plasma” (LOINC 14646-4) for female patients (n=1848, left) and male patients (n=5026, right) aged 60 to 65 years.

Using the *BioRef* platform, it is possible to infer RIs for previously underrepresented patient populations in RI studies. For instance, RIs for “HDL cholesterol” (LOINC 14646-4) in male and female clinical patients aged 60 to 65 years were estimated. The resulting RIs (with 90% CIs) and the accompanying histograms were generated on the fly and visible in the web applications (Figure 5).

The estimated RIs for female patients are 0.54 (90% CI 0.51-0.56) to 2.47 (90% CI 2.42-2.51) mmol/L and for male patients are 0.52 (90% CI 0.51-0.53) to 1.92 (90% CI 1.89-1.93) mmol/L, derived from the local population. These results are comparable to those from a published RI study that used similar routine clinical data, the same analytical system (Roche Cobas 8000), and similar laboratory data mining techniques for the estimation of locally specific RIs (female patients: 0.72, 90% CI 0.50-0.80, to 2.02, 90% CI 1.83-2.09 mmol/L; male patients: 0.54, 90% CI 0.49-0.65, to 1.30, 90% CI 1.24-1.63 mmol/L) [59]. Although these RIs do not fully overlap, they are locally significant and stratified by age, in contrast to other published RIs. It is established that high-density lipoprotein decreases with age and addressing this often missing age stratification is crucial [60,61]. This example highlights the need for adapted target ranges that take into account the specific condition of the patient based on their risk and value distribution [11].

### User Evaluation

During a follow-up project of *Swiss BioRef* (“BioRef - TI4Health*”)*, Inselspital; CHUV; and Tune Insight, which is a spin-off of the Swiss Federal Institute of Technology Lausanne, collaborated to deploy and evaluate the *TI4Health* system. Reviewers from both the clinical side and clinical data science were onboarded for a preliminary evaluation of the deployed platform to assess its variable accessibility, usability, and performance. Users expressed appreciation for the easy and streamlined web application GUI that quickly filtered their population of interest. Maintaining a perfect balance between a streamlined and intuitively usable GUI and a GUI that entails a complex query selection process is a challenging task. Managing this is crucial because the growing and progressively varying user base will make it even more challenging to anticipate future requirements. Query execution time has emerged as a potential issue for federated systems. Notably, data processing under homomorphic encryption does not cause delays, but rather the i2b2 format is the bottleneck in terms of performance. Using other data formats for which *TI4Health* offers additional connectors will alleviate this problem.

## Discussion

### Principal Finding: Federated Analytics Architecture

With the *BioRef platform* for federated confidential computing, an interoperable and secure framework for processing distributed multidimensional laboratory data from various cohorts forming a “big data” resource of laboratory measurements has been created and deployed for the first time in an operational setting. The use of a federated analytics approach allows the indirect provision of nonanonymized (ie, identifiable) patient data to a multicentric effort, which is, under the current data protection act, an almost impossible administrative task to tackle [34]. As *sensitive data* themselves are not shared between participating parties, the *BioRef approach* is compliant with both national and international data provision laws (ie, the European Union’s General Data Protection Regulation [GDPR]) [62]. Notably, the use of a distributed analytics system as deployed can significantly reduce the governance overhead for future multicohort collaborations [36]. It will also facilitate obtaining permission from ethical boards, as identifying information is retained only by the respective hospital.

### Harmonizing Data Resources

The differing data management systems and formats at individual clinical data warehouses are a limiting factor for smooth data provision; significant efforts are required to harmonize the data contribution of all data providers and ensure interoperability before the entry of the data into the *BioRef* infrastructure. For example, the implementation of LOINC on a national level has advanced notably over the last few years but still requires serious effort to provide high-quality metadata and quality control for laboratory analyses [63]. However, these standardization efforts are not only beneficial for the scope of this project but are also essential for the ongoing digital transformation of laboratory medicine, especially in the age of machine learning and artificial intelligence [64]. Clearly coded, high-dimensional laboratory data can essentially contribute to clinical research in the age of personalization [65]. With increasing data sizes made available for clinical research projects, clear ethical guidelines for “big data” research need to be established [66].

### Targeted RIs for Precision Medicine

Standard RIs are inferred under the assumption that an appropriate reference population can be defined as representing a “general” health status, either through a priori or a posteriori selection [67]. It is assumed that the only observed variation in the selected reference values stems from biological interindividual variation [68]. The use of newer methodologies allows the indirect estimation of RIs from real-world data that are considered a mixture of “pathological” and “nonpathological” values via various resolution techniques [69]. However, in the clinical context, where a variety of patient factors are considered during the physician’s anamnesis, RIs estimated from generally “non-pathological” reference individuals are seemingly not the most appropriate reference to compare patients’ blood test results with [12]. Especially in older patients, the differentiation between “disease” and the aging process is difficult; a functional decline observed in old age can originate both from a disease or the aging process itself. The differentiation can be made using peptide biomarkers (eg, N-terminal pro-B-type natriuretic peptide [70,71]), hormones (eg, thyroid-stimulating hormone [72,73]), and lipids (high-density lipoprotein cholesterol [60,61]). Age-related health concerns become prominent in aging populations, and appropriate “reference values” should comprise both values reflecting physiological changes and an increasing fraction of values that would generally be considered pathological to reflect the patient population [73]. Rather than trying to create RIs as “normal ranges” for aging populations, these “expectations ranges” help evaluate the specific patient’s test result in the appropriate context of similar patients (“digital twins”). The possibility to include and exclude specific diagnoses allows the adjustment and fine-tuning of these expected ranges to a variety of multimorbid complexes (eg, diabetes, hyperlipidemia, coronary heart disease, or renal impairment). Here, we suggest that being able to map additional patient parameters such as age and sex as well as individual combinations of multiple morbidities on the analysis of locally derived RIs can essentially provide *personalized target ranges* fit for application in precision medicine. With the interactive GUI of the web client, these *targeted RIs* can be generated on the fly, which can then be effectively used when paired with established RIs. Although these are not “personalized” RIs per se, that is, referring to a single patient of interest, they provide second-level information regarding the particularities of a patient group of interest [74]. In cases where there are no RIs established locally for a particular age and sex group, these personalized target ranges can serve as a useful substitute.

### Strengths and Limitations

Despite the many benefits that a decentralized data-sharing system offers, a stringent quality control step of centralized data alignment is missing. Therefore, local quality control at all participating sites following a standardized protocol, as well as establishing trust among collaborating partners for the continuation of data provision to the system, is a must. The basis for the overall BioRef data set is the local population, and a broad spectrum of diagnoses is covered in the data set. Very specific diagnoses (eg, psychiatric disorders) or complex combinations of diagnoses may still be underrepresented or even missing; however, this may be overcome in the future through the inclusion of specialized hospitals, broadening the data basis. Mutually beneficial collaborations between additional national and international hospitals and data providers are, therefore, encouraged. Although a centralized approach ensures easily verifiable results for testing and validation (each data holder has full access to the underlying data set), a federated approach allows the onboarding of institutions that are not willing to share data in a centralized setting. This allows for insights from more data than each individual data provider holds. The motivation and deployment conditions for federated and centralized approaches are slightly different, and their applicability depends on the context of the project. An in-depth comparison, scalability in a multinational context, and applicability in the clinical context will need to be addressed in a follow-up study, as these are beyond the scope of this pilot project presenting the first federated setup for RI estimation.

Another challenge for any international network is mirroring the ethnic diversity found in patients across countries, which can influence RIs [33]. The data should include information on the ethnic background of a patient, which needs to be gathered by hospitals. However, this information may not be routinely collected. Preanalytics, for example, sample collection or handling, are another factor that may vary between countries and may hamper data interoperability. Providing additional metainformation on the preanalytics akin to the implemented information on analyzer and reagent may be the way forward, for example, using Standard PREanalytical Codes [75].

### Comparison With Prior Work

Multicenter studies operating under a common and centralized standardization effort have already aimed at estimating country-specific RIs [31,76], and previous studies have leveraged routine laboratory data to assess population-specific RIs to some extent [32,33]; however, to our knowledge, a federated query system has not been implemented so far. Although the Canadian Laboratory Initiative on Pediatric Reference Intervals and the Pediatric Reference Intervals Initiative in Germany provide RIs for laboratory analytes in pediatrics via interactive web applications, they both rely on the centralization of the data source [31,76]. The clear advantage of a federated approach, such as BioRef-TI4Health, is that hospitals can contribute data to evaluation without actually sharing them. In the era of “big data,” where an increasing amount of health data is available, this is especially useful, as full anonymization of sensitive data (ie, health data) can be difficult to attain [77].

The use of homomorphic encryption in addition to data aggregation adds an additional layer of security: several publications have shown that aggregated data have the potential to reveal information about individuals (eg, membership in a sensitive cohort and undisclosed private or sensitive attributes) through statistical inference even if the data themselves do not directly identify specific persons [78-80]. Users can only decrypt and see the result of the aggregation of each individual site’s response to the query. Unencrypted setups for remote federated analysis [81,82] cannot fulfill these requirements. In addition, the use of homomorphic encryption to protect site-level aggregated data helps comply with the “data minimization” principle (GDPR article 5) by revealing only the information that is needed for the user’s purposes. Moreover, it satisfies the “privacy by design” principle (GDPR article 25) by minimizing the risk that arises from personal data breaches by making personal information unintelligible to anyone not authorized to access it.

Several different approaches for federated analytics have been implemented and applied to medicine, starting from off-the-shelf federated learning to advanced alternatives such as swarm learning [83-89]. However, most of the time, these approaches were limited to project-specific demonstrations and are not yet implemented in clinical operational settings through scalable and sustainable infrastructures. Examples of successful infrastructure implementations are the Accrual to Clinical Trials Network, TriNetx, and Clinerion [90-92]. However, none of them are particularly focused on laboratory medicine, and BioRef-TI4Health stands out by using state-of-the-art, advanced, and privacy-enhancing technologies to protect data and patient privacy. It will be interesting to compare published RIs on a broad scale with our cohort-specific target ranges in a follow-up study.

### Conclusions and Outlook

Within the scope of the *Swiss BioRef* project, a privacy-preserving federated computing network accessible via a web-based GUI has been established. With *BioRef*, the SPHN’s long-term goal of transforming medicine toward precision and personalization has reached one of its first manifestations [93]. It allows physicians and clinical researchers to map the individual complexity of their patients to a rich multicohort data pool and permits a substantiated statistical analysis to infer precise and highly relevant RIs. The federated nature of the approach together with the implemented cryptographic mechanisms helps release the brakes which legislation and local data-sharing policies may at times represent to research and related ambitious projects. The federated setup will also facilitate a potential extension of the network, potentially on an international level.

Long-term sustainability is a widespread problem in academic projects, as the costs of both infrastructure operation and maintenance must be addressed. Here, the open architecture and simplified onboarding process of the BioRef platform offer a chance to include academic partners, professional clinical medicine societies, and the diagnostics industry. Tune Insight maintains the Swiss BioRef TI4Health codebase, provides support, and performs further customization for the future of BioRef.

Collaboration with a broad spectrum of stakeholders is fundamental to the continuation of the *Swiss BioRef* project. It is important not only to showcase the relative ease of use of the proposed platform to both health professionals and clinical researchers who could be potential new end users but also to establish trust regarding the novelty of the developed infrastructure of multicohort data sharing. A stakeholder dialogue could inform novel guidelines for specific health conditions that have applications in the clinical context, which could benefit the harmonization of both the estimation and use of RIs across multiple cohorts. Collaboration with the international Task Force on Global Reference Interval Database of the IFCC is currently being promoted to implement an international system for RI estimation [13].

Given the modularity of both the *BioRef* consortium and the *BioRef-TI4Health* system architecture (future national and international partners can join with relative ease) as well as the applications (extendable for additional types of statistical analyses or variables), we see a bright future for *personalized target ranges* in Switzerland and beyond.

## Appendix

Multimedia Appendix 1 The full list of the 46 laboratory variables, uniquely defined by Logical Observation Identifiers Names and Codes (LOINC) encoding, for which quantitative laboratory test results (“measurements”) were collected from each contributing data cohort individually.

## Abbreviations

CHUV: Centre Hospitalier Universitaire Vaudois
CLSI: Clinical and Laboratory Standards Institute
GDPR: General Data Protection Regulation
GM: German Modification
GUI: graphical user interface
i2b2: Informatics for Integrating Biology and the Bedside
ICD: International Classification of Diseases
IFCC: International Federation of Clinical Chemistry
LOINC: Logical Observation Identifiers Names and Codes
RI: reference interval
SPHN: Swiss Personalized Health Network
