# Correction: The BioRef Infrastructure, a Framework for Real-Time, Federated, Privacy-Preserving, and Personalized Reference Intervals: Design, Development, and Application

**DOI:** 10.2196/54809

**Published:** 2023-12-07

**Authors:** Tobias Ueli Blatter, Harald Witte, Jules Fasquelle-Lopez, Christos Theodoros Nakas, Jean Louis Raisaro, Alexander Benedikt Leichtle

**Affiliations:** 1 University Institute of Clinical Chemistry University Hospital Bern Bern Switzerland; 2 Graduate School for Health Sciences University of Bern Bern Switzerland; 3 Biomedical Data Science Center University Hospital Lausanne University of Lausanne Lausanne Switzerland; 4 Laboratory of Biometry University of Thessaly Volos Greece; 5 Center for Artificial Intelligence in Medicine University of Bern Bern Switzerland

In “The BioRef Infrastructure, a Framework for Real-Time, Federated, Privacy-Preserving, and Personalized Reference Intervals: Design, Development, and Application” (J Med Internet Res 2023;25:e47254) the authors made one addition and one removal.

The authorship was previously published as:

Tobias Ueli Blatter^1,2*^, MSc; Harald Witte^2*^, PhD; Jules Fasquelle-Lopez^3^, MSc; Jean Louis Raisaro^3*^, Prof Dr; Alexander Benedikt Leichtle^1,4*^, Prof Dr Med

The following author, ORCID, and associated affiliations have been added in the fourth position of the authorship:

Christos Theodoros Nakas^1,4^, Prof Dr (ORCID ID: 0000-0003-4155-722X)

University Institute of Clinical Chemistry, University Hospital Bern, Bern, Switzerland

Laboratory of Biometry, University of Thessaly, Volos, Greece

The authorship and affiliations have been adjusted accordingly, and will now read as follows:

Tobias Ueli Blatter^1,2*^, MSc; Harald Witte^2*^, PhD; Jules Fasquelle-Lopez^3^, MSc; Christos Theodoros Nakas^1,4^, Prof Dr; Jean Louis Raisaro^3*^, Prof Dr; Alexander Benedikt Leichtle^1,5*^, Prof Dr Med

Additionally, the new author’s mention will be removed from the first paragraph of the Acknowledgments, which previously appeared as:

The Swiss BioRef project was funded by the Swiss Personalized Health Network (2018DEV22), the University Hospital Bern, and Swiss Paraplegic Research. The Swiss BioRef project was led by a computational medicine group in Bern, which developed the necessary IT components in collaboration with the health informatics and data privacy group in Lausanne. The authors would like to thank their collaborators; Simon Le Bail-Collet; their partners from BioMedIT, DCC, SIB, and Unitecta; Christos T Nakas for continued support; and, most importantly, all the patients who provided written consent for their data to be used in the study. The authors are indebted to Frédéric Erard, Julia Maurer, and Jana Rochlitz from SIB, DCC, and Insel, respectively, for their substantial support in establishing the BioRef consortium agreement.

And will now be changed to:

The Swiss BioRef project was funded by the Swiss Personalized Health Network (2018DEV22), the University Hospital Bern, and Swiss Paraplegic Research. The Swiss BioRef project was led by a computational medicine group in Bern, which developed the necessary IT components in collaboration with the health informatics and data privacy group in Lausanne. The authors would like to thank their collaborators; Simon Le Bail-Collet; their partners from BioMedIT, DCC, SIB, and Unitecta; and, most importantly, all the patients who provided written consent for their data to be used in the study. The authors are indebted to Frédéric Erard, Julia Maurer, and Jana Rochlitz from SIB, DCC, and Insel, respectively, for their substantial support in establishing the BioRef consortium agreement.

The correction will appear in the online version of the paper on the JMIR Publications website on December 7, 2023 together with the publication of this correction notice. Because this was made after submission to PubMed, PubMed Central, and other full-text repositories, the corrected article has also been resubmitted to those repositories.

